# A bench-to-bedside study about trigger asynchronies induced by the introduction of external gas into the non-invasive mechanical ventilation circuit

**DOI:** 10.1038/s41598-021-03291-y

**Published:** 2021-12-10

**Authors:** Cristina Lalmolda, Pablo Flórez, Carles Grimau, Roberto Larrosa, Marta Corral, Javier Sayas, Manel Luján

**Affiliations:** 1 Servei de Pneumologia. Hospital Universitari Parc Taulí, Carrer Parc Taulí s/n, 08208 Sabadell, Barcelona Spain; 2 Servicio de Neumologia. Hospital, Universitario Príncipe de Asturias, Alcalá de Henares, Madrid, Spain; 3 Servicio de Neumologia, Hospital Universitario 12 de Octubre, Madrid, Spain; 4 Centro de Investigación Biomédica en Red Enfermedades Respiratorias, CIBERES, Bunyola, Spain

**Keywords:** Physiology, Medical research

## Abstract

Treatments that require the introduction of external gas into the non-invasive ventilation (NIV) circuit, such as aerosol and oxygen therapy, may influence the performance of the ventilator trigger system. The aim of the study was to determine the presence and type of asynchronies induced by external gas in the NIV circuit in a bench model and in a group of patients undergoing chronic NIV. Bench study: Four ventilators (one with two different trigger design types) and three gas sources (continuous flow at 4 and 9 l/min and pulsatile flow at 9 l/min) were selected in an active simulator model. The sensitivity of the trigger, the gas introduction position, the ventilatory pattern and the level of effort were also modified. The same ventilators and gas conditions were used in patients undergoing chronic NIV. Bench: the introduction of external gas caused asynchronies in 35.9% of cases (autotriggering 73%, ineffective effort 27%). Significant differences (p < 0.01) were detected according to the ventilator model and the gas source. In seven patients, the introduction of external gas induced asynchrony in 20.4% of situations (77% autotriggering). As in the bench study, there were differences in the occurrence of asynchronies depending on the ventilator model and gas source used. The introduction of external gas produces alterations in the ventilator trigger. These alterations are variable, and depend on the ventilator design and gas source. This phenomenon makes it advisable to monitor the patient at the start of treatment.

## Introduction

Non-invasive ventilation (NIV) is widely used in the treatment of acute^1^ and chronic respiratory failure, and is mainly administered for night-time ventilatory support^2^.

NIV can be used in isolation or combined with other treatments, which may require external devices that introduce variable amounts of gas mixture into the circuit. Aerosol medications are commonly used in the treatment of respiratory tract diseases^3^. They offer certain advantages over non-inhalation therapy, one being that they minimize toxicity by providing higher concentrations of the drug in the airway while low systemic concentrations are maintained^4^ Aerosolized treatment is used relatively frequently with NIV, mainly to administer bronchodilator drugs and/or antibiotics^5^. In an acute situation, the suspension of NIV in order to administer these treatments may entail risks for the patient^4,6^.

The efficacy of aerosol therapy in patients undergoing NIV has been studied in terms of drug administration and also factors regarding the device (i.e., the position and type of nebulizers)^3,7–13^. Regarding the implications for the ventilator, it is known that the administration of external gas can influence the patient’s tidal volume and patient-ventilator synchronization^14^. As a consequence, it may be necessary to adjust the ventilator mode and parameters when providing aerosol therapy for patients on mechanical ventilation^7,15,16^.

Another clinical situation that may require the introduction of external gas into the ventilation circuit is the need to provide supplemental oxygen at variable flows, usually lower than the nebulization flow. Many of the ventilators used for NIV are not equipped with an oxygen mixer or specific ports for the administration of supplemental oxygen; therefore, supplemental oxygen is usually administered by adding it to the mask or the circuit^11^. This external flow may have an effect on the performance of NIV, especially its trigger function, although this effect has not been studied to date^17^.

Thus, our hypothesis was that, when an external flow (of oxygen) was introduced in the circuit, it will impair the trigger function of the ventilator, as it modifies the trigger threshold for flow changes.

The aim of the present study was to evaluate the presence of trigger asynchronies resulting from the introduction of an external gas into the circuit during NIV in two settings:

- In a bench study, under different ventilatory patterns (obstructive or restrictive), sensitivity levels, levels of patient effort^18^ and different sources, quantities and locations of the gas introduced (continuous versus pulsatile flow compressor)
- In a short clinical study in patients receiving chronic NIV, in which the bench model previously described is reproduced.

## Methods

### Design of the bench study

The experiment was performed under simulated conditions in the NIV laboratory. A breathing simulator (series 1101, Hans Rudolph, Inc., Shawnee, Kansas) was used to test the different ventilators, which were connected to the simulator through a single standard 2-m tube with a leak in the expiratory port (Model 5804000, Intersurgical España SL, Madrid) placed at the distal end of the ventilator. The leak port was maintained throughout the experiment to simulate the intentional leak used in clinical practice in NIV with a single-limb configuration.

For the introduction of the external gas, a T-piece with a 3.5-mm diameter side port (Model 2713000. Intersurgical España SL, Madrid) was placed proximally to the ventilator first and then distally to the leak.

**Figure 1:**
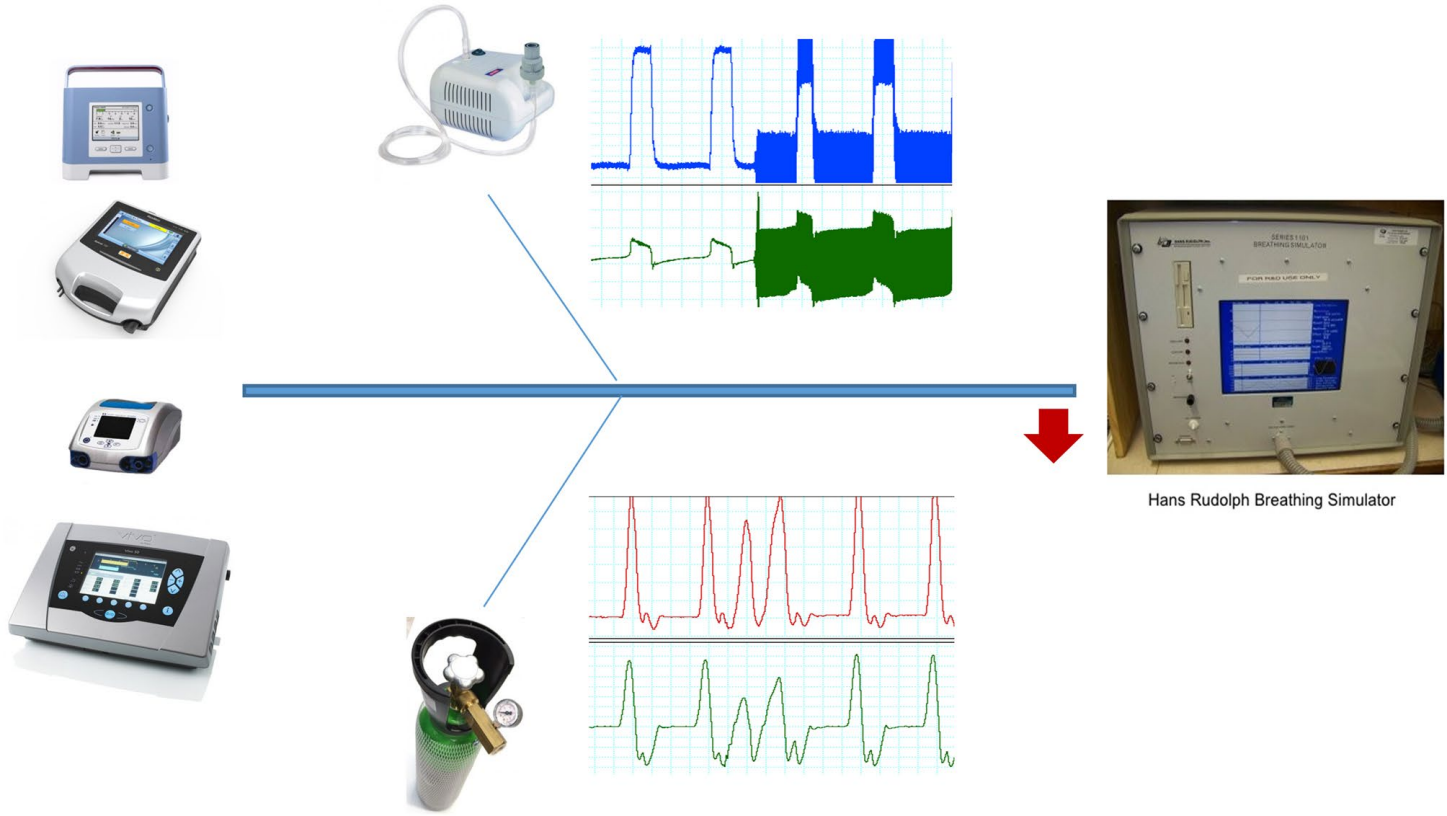
Bench study setup.

Three different treatment modalities were added to the NIV set-up:

- In the first model, a compressor (Mini Plus, APRES MEDICAL SL, Spain) was connected to a tube in the circuit. The flow of the compressor was monitored prior to placement, and a value of 9 l/min was obtained (Fig. 1).
- The previous model was reproduced in terms of the amount of gas introduced, but a continuous gas source with a pressure reducer and flowmeter (compressed air bottle, Nippon Gases, Madrid) was used.
- Finally, in the third model, a continuous gas source was used at 4 l/min. Medical air was used to avoid oxidation of the simulator parts due to the use of pure oxygen.

The simulator was programmed as follows: for lung mechanics variables, compliance was set at 30 ml/cm H2O and resistance was set at 5 cm H_2_O/l/s in a restrictive pattern and at 60 ml/cm H_2_O and 18 cm H_2_O/l/s respectively in an obstructive pattern (21). To simulate patient effort, the amplitude was programmed at 6 cmH_2_O for low effort and 14 cmH_2_O for high effort, depending on the condition simulated. Finally, the frequency of spontaneous breathing was established at 15 breaths/min.

**Figure 2:**
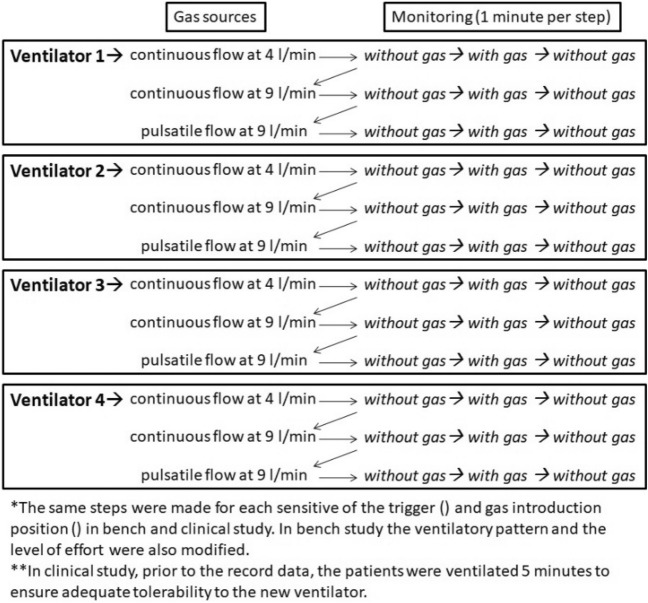
Protocol for testing through different steps.

Four commercial ventilators (one with two types of triggers) with the same pressure support values (IPAP 15 cm H_2_O, EPAP 5 cm H_2_O) were compared: (1) Vivo 50 (General Electric, Sweden), (2) Astral 150 (ResMed, Australia), (3) Trilogy 100 (Philips Respironics, Pennsylvania), and (4) Puritan Bennett (PB) 560 (Covidien, Massachusetts). The parameters selected for each ventilator are summarized in Table 1. Figures 1 and 2 explain the settings and protocol of testing.

**Table 1:** Parameterization of the ventilators in the bench study model.

Model	PS	Inspiratory trigger			Cycling off criterion			Ramp		RF^e^
		S^b^	M^b^	NS^b^	S^c^	M^c^	L^c^	O^d^	R^d^	
Trilogy	10^a^	2 lm	5 lm	9 lm	60%	50%	30%	2	3	12
Trilogy AutoTrak	10^a^	Automatic			Automatic			2	3	12
Vivo 50	10^a^	2	4	7	7	4–5	2	2	4	12
Astral 150	10^a^	High	Medium	low	High	Medium	Low	150 ms	250 ms	12
PB 560	10^a^	2	4	6	60%	50%	30%	2	3	12

Trigger setups for Astral, VIVO and PB 560 are indicated in an analogic scale.

^a^IPAP: 15; EPAP: 5.

^b^Inspiratory trigger: S: Sensitive; M: Moderately sensitive; NS: Not sensitive.

^c^Cycling off criterion: S: Short; M: Medium; L: Long.

^d^Pulmonary mechanics: O: Obstructive, R: Restrictive.

^e^Respiratory frequency.

Signal acquisition system: an external polygraph (16Sp Powerlab, AD Instruments, Australia) equipped with two pressure transducers (model 1050) and two pneumotachographs (S300, instrumental dead space = 70 ml, resistance = 0.0018 cm H_2_O/l/s) was used. The sampling frequency was adjusted to 200 Hz, and the polygraph was connected to a personal computer equipped with Chart 7.0 software for Windows.

The ventilation periods were 1 min for each step (without gas → with gas → without gas). This sequence was performed twice in each group of established conditions. The gas-induced asynchronies were compared by interpreting the graphs of pressure and flow time according to the SOMNONIV algorithm, by three independent observers^19^. Combinations of effort, trigger, and lung mechanics that induced asynchronies without the need of gas introduction were eliminated from the subsequent analysis.

### Design of the short clinical study

The study was conducted with patients from the Pneumology Service of the Corporació Sanitària Parc Taulí (Sabadell, Barcelona) who met the following criteria: age over 18, hospital admission for acute on chronic respiratory failure, home NIV (single-limb system with intentional leakage) and more than 6 months of use with adequate compliance (5 h/night or more). Patients with underlying psychiatric disease were excluded. The study was conducted during the patient's predischarge phase (the same day or the day before). Patients after a COVID-19 admittance were excluded (all of the included patients had a negative swab test PCR at admission). Due to the epidemiological status of the area, a short period of time between waves was employed.

**Figure 3:**
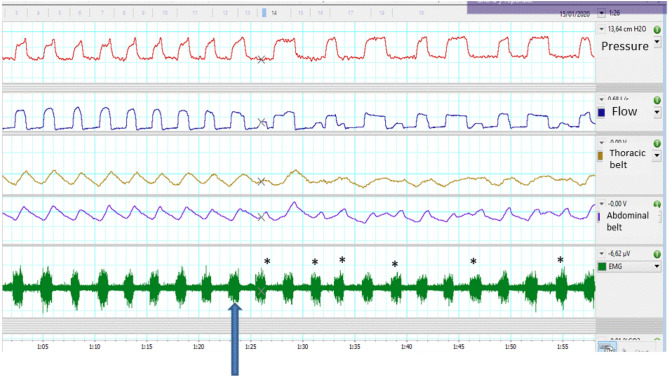
Example of a fully monitored patient. After the gas introduction (arrow), ineffective efforts (asterisks) can be easily detected. Observe the unassisted efforts in the belts and EMG signals.

Protocol: The procedure was performed in the patient's room during his or her hospital stay to avoid additional visits. The patient was placed in the supine position, his own home NIV interface was placed in a single-limb system, along with the commercial ventilator to be studied. A small period for adaptation (5–10 min), if the ventilator tested was different from their home ventilator, was employed, to ensure comfort. The same ventilators tested in the bench model were evaluated at the bedside in random order. The parameters of the ventilator were the same as those that the patients used at home (that is, unlike the bench study, the sensitivity of the trigger was not modified). The monitoring system was essentially the same as previously described, with the incorporation of thoracic and abdominal bands and parasternal electromyography to better evaluate asynchronies, in addition to pulse oximetry control. As the introduction of external gas could cause distortion in the pressure and flow waveforms leading to misclassification of asynchronies, it was helpful the use of monitoring tools to assess patient’s ventilatory pattern. Figure 3 reflects an example of a fully monitored patient. The monitoring was prepared before the experiment and maintained until the ventilation was withdrawn. The gas sources were the same as those used in the bench study model. Monitoring of leakage was performed during clinical study through ventilator screen to detect sudden changes in non-intentional leakage that could eventually induce asynchronies. Sequences with changes > 10% in nonintentional leakage were discarded and repeated. All methods were performed in accordance with the relevant guidelines and regulations. The study was approved by the hospital ethics committee of “Comité de ética e investigación con medicamentos (CEIm) del Parc Taulí de Sabadell” (ref. 2019/511). Written informed consent was obtained in the day of the experiment. Trial was registered at Clinicaltrials.gov (NCT04884828).

### Statistical analysis

Quantitative data are expressed as means and standard deviation (µ, sd) according to the normality of the variables, which was assessed using the Shapiro Wilk test. Qualitative data are expressed as absolute frequencies. The chi-square test or Fisher's exact test was used in the absence of normality to evaluate the presence or absence of gas-induced asynchrony by comparing the variables controlled in the bench study and the ventilator model, gas source, and interindividual variability in the clinical study. Bonferroni’s adjustment for multiple chi-square testing was used. The level of significance was established at p < 0.05. Accepting an alpha risk of 0.05 and a beta risk of 0.2, seven subjects were necessary to recognize a significant difference, consisting in the absence of asynchronies before gas and appearance after gas, considering a drop-out rate of 20%.

### Ethics approval and consent to participate

The study was approved by the hospital ethics committee “Comité de ética e investigación con medicamentos (CEIm) del Parc Taulí de Sabadell” (ref. 2019/511). Written informed consent was obtained.

## Results

### Bench study model

A total of 312 simulation sequences were obtained after combining the different variables (gas source, ventilatory pattern, position of entry into the circuit, trigger sensitivity and patient effort). In 67 cases (21.4%), asynchrony under basal conditions was documented; these were therefore excluded from the final analysis, which included 245 combinations.

Table 2 shows the differences between ventilators in terms of percentages and types of baseline asynchronies in the traces that were not included in the analysis.

**Table 2:** Distribution between ventilators of the simulation sequences in which there was baseline asynchrony (p < 0.01 among ventilators, chi-square test).

	Type of basal asynchrony, if present			Total
	Without asynchrony	Autotrigger	Ineffective effort	
**Ventilator model**				
Trilogy	60 (83.3%)	12 (16.7%)	0	72
Trilogy AutoTrak	23 (95.8%)	1 (4.2%)	0	24
Vivo 50	50 (69.4%)	20 (27.7%)	2 (2.8%)	72
Astral 150	70 (97.2%)	1 (1.4%)	1 (1.4%)	72
PB 560	42 (58.3%)	22 (30.6%)	8 (11.1%)	72
Total	245 (78.5%)	56 (17.9%)	11 (3.5%)	312

The introduction of gas induced asynchronies in 35.9% (88/245) of the simulations. Autotrigger was the most frequent asynchrony (64/88), followed by ineffective effort (24/88). In 56/88 cases, the gas-induced asynchrony remained unchanged (without correction) throughout the period of gas introduction. Ineffective effort was corrected in a significantly higher percentage of situations (16/24) than autotrigger (16/64, p < 0.01).

Regarding the factors conditioning asynchrony, statistically significant differences were found in the presence of asynchronies between ventilators, type of gas (pulsatile vs continuous), gas flow (4 vs 9 l in continuous flow), level of effort of the simulator, ventilatory pattern and trigger sensitivity. Table 3 shows the results, differentiated by the asynchronies induced.

**Table 3:** Percentage and type of asynchronies induced by the introduction of external gas as a function of the variables controlled in a bench study model.

	No asynchrony	Autotriggering	Ineffective effort	Total	p value*
**Ventilator**					
Trilogy	50 (83.3%)	1 (1.7%)	9 (15%)	60	< 0.01
Trilogy (AutoTrak)	19 (82.6%)	1 (4.3%)	3 (13.1%)	23^a^	
Vivo 50	24 (48%)	22 (44%)	4 (8%)	50	
Astral 150	47 (67.1%)	17 (24.3%)	6 (8.6%)	70	
PB 560	17 (40.5%)	23 (54.8%)	2 (4.7%)	42	
**Type and source of gas**					
Compressor at 9 l/min	30 (37.5%)	44 (55%)	6 (7.5%)	80	< 0.01
Continuous gas 9 l/min	54 (63.6%)	15 (17.6%)	16 (18.8%)	85	
Continuous gas at 4 l/min	73 (91.2%)	5 (6.3%)	2 (2.5%)	80	
**Pattern**					
Obstructive	73 (58.8%)	30 (24.2%)	21 (17%)	124	< 0.01
Restrictive	84 (69.4%)	34 (28.1%)	3 (2.5%)	121	
**Gas position**					
Proximal to the ventilator	69 (57.5%)	39 (32.5%)	12 (10%)	120	ns
Distal to the ventilator	88 (70.4%)	25 (20%)	12 (9.6%)	125	
**Effort of simulator**					
High	91 (74%)	31 (25.2%)	1 (0.8%)	123	< 0.01
Low	66 (54.1%)	33 (27%)	23 (18.9%)	122	
**Trigger sensitivity**					
Sensitive	27 (61.4%)	15 (34.1%)	2 (4.5%)	44	0.01
Intermediate	57 (62%)	29 (31.5%)	6 (6.5%)	92	
Not very sensitive	54 (62.8%)	19 (22.1%)	13 (15.1%)	86	
AutoTrak	19 (82.6%)	1 (4.3%)	3 (13.1%)	23^a^	

*Chi-square test.

^a^The number of simulations under these conditions was lower because a single level of sensitivity was used in some of the situations.

After gas closure, asynchronies were induced in 101 situations (96 with autotriggering), and the vast majority (95 in total) were transient. Only in six situations was the autotriggering constant until the end of the phase.

### Short clinical study

Ten patients were initially selected, one was unable to complete the protocol and two were excluded due to a poor signal quality of the EMG. Seven patients were finally included in the study in the short validation cohort. Their characteristics are detailed in Table 4, which also presents the most relevant ventilator parameters.

**Table 4:** Characteristics and devices of the patients included in the clinical study.

Num	Sex (m, f)	Age (years)	Pathology	PS	Trigger	Cycle	Ventilator	Interphase
1	M	70	COPD	13	Moderately sensitive	Medium	LUMIS	Oronasal
2	M	64	Restrictive	10	Sensitive	Medium	LUMIS	Oronasal
3	F	61	Neuromuscular	15	Sensitive	Long	ASTRAL	Oronasal
4	M	78	COPD	12	Moderately sensitive	Short	LUMIS	Oronasal
5	M	64	COPD	12	Sensitive	Short	TRILOGY	Oronasal
6	M	65	Restrictive	12	Moderately sensitive	Long	STELLAR	Oronasal
7	M	63	COPD	12	Moderately sensitive	Medium	LUMIS	Oronasal

After the introduction of external gas, new-onset asynchronies occurred in a total of 22/105 situations (20.4%).

The most prevalent asynchrony was autotrigger, in 17/105 tested situations, followed by ineffective effort (5/105). No correction of asynchrony was observed during the period of gas administration. As Table 5 shows, and as in the bench study model, there were significant differences depending on the type of gas and ventilator model used.

**Table 5:** Percentages and types of asynchronies induced by the introduction of external gas as a function of the variables controlled in the clinical model.

	No asynchrony	Autotriggering	Ineffective effort	Total	p value*
**Ventilator**					
Trilogy	19 (90.5%)	2 (9.5%)	0	21	< 0.01
Trilogy (AutoTrak)	21 (100%)	0	0	21	
Vivo 50	10 (47.6%)	6 (28.6%)	5 (23.8%)	21	
Astral 150	19 (90.5%)	2 (9.5%)	0	21	
PB 560	14 (66.6%)	7 (33.3%)	0	21	
**Type of gas**					
Continuous gas 4 l/min	33 (94.2%)	1 (2.9%)	1 (2.9%)	35	< 0.05
Continuous gas 9 l/min	28 (80%)	5 (14.3%)	2 (5.7%)	35	
Compressor at 9 l/min	22 (62.8%)	11	2	35	

*Chi-square test.

## Discussion

The main findings of the study are the following. In the bench study, the introduction of external gas to the circuit in NIV was associated with trigger asynchronies in 36% of the situations; autotrigger was the most frequently occurring asynchrony, followed by ineffective effort. In addition, the latter showed a greater degree of spontaneous correction during gas introduction. The occurrence of asynchronies was related to the different ventilators studied, the source and flow of the gas, and lung mechanics. The results of the clinical study also corroborated a significant increase in the number of asynchronies, but in a significantly lower proportion than in the bench study. However, the conditioning factors of the asynchronies (pulsatile flow use and trigger design) were essentially the same as those found in the bench study.

The design of the trigger function of the ventilators used for NIV has undergone significant improvements, always in search of a greater sensitivity (i.e., triggering against decreasing efforts of the patient) and trying to preserve specificity (i.e., avoiding unwanted triggering in the absence of effort). This objective has led to significant differences in design between manufacturers, which may help to explain the findings of this study. Some of the ventilators studied (Astral, Trilogy, PB 560) are equipped with flow trigger systems, which is probably the most frequently used design at present^20^. These systems have shown greater sensitivity than the old pressure trigger models^21^. However, the use of flow thresholds with fixed values for triggering can cause problems in the presence of leaks if the ventilator’s leakage compensation flow is higher than its trigger threshold (which might be misinterpreted as patient effort). For this reason, flow trigger systems are often accompanied by an algorithm that can be used to automatically adjust their sensitivity as a function of leakage^22^. These algorithms operate independently of the ventilator’s pre-set sensitivity level, but they can lead to paradoxes such as decreased trigger sensitivity in the presence of leaks, which may eventually favour the occurrence of ineffective effort.

To compensate for these drawbacks, alternative (but more complex) designs, such as AutoTrak^®^ (Philips Respironics), have been devised based on the crossing points that are generated by a delayed virtual waveform and superimposed (using the flow waveform method) on the native waveform^23^ or are derived from a calculation based on the so-called *trigger energy* (Breas) of the first derivative of the flow^24^. In general, these more complex systems tend to be more sensitive but have a greater tendency towards automatic triggering^23^. These differences in design, together with the levels of trigger sensitivity specific to each manufacturer, can explain the differences in the behaviour of the ventilators analysed in this study, even under basal conditions.

Another factor that generated asynchronies was the amount of gas introduced into the system. Aerosol therapy generally requires a minimum gas flow of 8 l/min. This continuous flow contributes to the pressure level that the ventilator reaches during the respiratory cycle and causes a substantial decrease in the flow administered by the ventilator itself^7^. This decrease also automatically modifies the sensitivity of the trigger in the flow models in a manner inverse to the previously described leakage model, and may favour the appearance of asynchronies. The nature of the mechanisms employed to correct trigger thresholds related to leak levels operate in very short timeframes. For example, Resmed uses a moving timeframe of 6 s, that may narrow to 3 s to adjust trigger threshold, marketed as *Vsync*^®^^22^ whereas other manufacturers employ a limited number of breaths to make any correction. Thus, all these systems operate in a very narrow time frame, under 10 s, allowing for very fast changes in the trigger threshold. This fact may explain why around 30% of initial asynchronies may correct spontaneously in the very next seconds. Finally, the greater number of asynchronies induced by the compressor at equal flow could be attributed to the wide fluctuations in flow generated by the device compared to the continuous flow of gas (see Fig. 1). It should be remembered that the operating mechanism of a compressor is based on the displacement of a piston on a cylinder, which generates a pulsatile flow.

Leak estimation is closely related to the algorithms that may correct trigger threshold in different leak leves. Some authors have evaluated the ability of different ventilators to compensate for leaks^25^ and have concluded that certain ventilators cannot guarantee the pre-established tidal volume when involuntary leaks are introduced, a phenomenon that they attribute mainly to the development of asynchronies (autotriggering) rather than to an inaccurate estimation of the tidal volume. Other bench studies have shown that the tidal volume estimation of home ventilators can be substantially modified by the presence of continuous or random leaks^25,26^. Anyway, leak estimation may play a relevant role in the way the ventilator modifies their triggering function in the presence or absence of flow input at the circuit level.

The findings of the present study have implications for clinical practice since they reflect situations (especially the use of compressors with certain ventilator models) that may require ventilator parameters to be modified in some cases.

Some limitations of this study should be considered. First, in both the bench and the clinical studies, the effects were examined for short, limited periods of time. Therefore, we do not know the compensation capacity of the ventilators over longer periods of time, although, based on the recalculation algorithms against leaks at level zero, these usually occur after only a few cycles. Second, the model was applied while the patient was awake; although this is when most similar interventions are performed, the supply of oxygen in the tubing is usually delivered over the long term, and we do not know whether the systematic use of supplemental oxygen is associated with a higher percentage of asynchronies at night.

Third, all of these ventilators are equipped with oxygen inlet ports. Obviously, mixing oxygen with the air previously to be driven by the turbine and sensed by the output flow sensor will not interfere with triggering option. But most of the low-level home care ventilators do not have built-in oxygen ports, and supplemental oxygen needs to be plugged in the circuit, via a t-piece. These homecare ventilators operate with the same algorithms for correcting triggering thresholds as the ones tested here, so results are fully applicable. Even more, to nebulise drugs in the circuit an oxygen input in the circuit is still needed, unless using ICU ventilators with synchronised, built-in flow sources. Finally, to avoid premature oxidation problems with the simulator, pressurized medical gas (21% oxygen accompanied by 78% nitrogen) was used. Under clinical conditions, 100% pure oxygen supplementation would have been used. The different densities of pure oxygen relative to air (relative density to air 1.1052) may modify the calibration, and the ventilator’s response to the flow is different from that evaluated in this study. The density (g/l) and viscosity (micropoises) of oxygen (1.429 and 192.6 respectively) are higher than those of air (1.293 and 170.8 respectively).

In conclusion, when an external gas is introduced into the circuit in NIV mode, the ventilators tested show different responses of asynchronies and different degrees of correction. These behaviours differ according to the amount of gas, the trigger level and the external gas source. These findings make it advisable to monitor, and possibly change, the parameters when certain ventilator models and applied gas sources are used.
